# Hepatic stellate cell activation markers are regulated by the vagus nerve in systemic inflammation

**DOI:** 10.1186/s42234-023-00108-3

**Published:** 2023-03-31

**Authors:** Osman Ahmed, April S. Caravaca, Maria Crespo, Wanmin Dai, Ting Liu, Qi Guo, Magdalena Leiva, Guadalupe Sabio, Vladimir S. Shavva, Stephen G. Malin, Peder S. Olofsson

**Affiliations:** 1 Department of Medicine Solna, Laboratory of Immunobiology, Division of Cardiovascular Medicine, Center for Molecular Medicine, Karolinska Institutet, Stockholm, Sweden; 2 Department of Biochemistry, Faculty of Medicine, Khartoum University, Khartoum, Sudan; 3 Department of Medicine Solna, Stockholm Center for Bioelectronic Medicine, Karolinska Institutet, Karolinska University Hospital, Stockholm, Sweden; 4 Spanish National Center for Cardiovascular Research (CNIC), Madrid, Spain; 5 Department of Immunology, School of Medicine, Complutense University of Madrid, Madrid, Spain; 6 Institute of Bioelectronic Medicine, Feinstein Institutes for Medical Research, Manhasset, NY USA

**Keywords:** Kupffer cells, Liver, Non-alcoholic fatty liver disease, PNPLA3

## Abstract

**Background:**

The liver is an important immunological organ and liver inflammation is part of the pathophysiology of non-alcoholic steatohepatitis, a condition that may promote cirrhosis, liver cancer, liver failure, and cardiovascular disease. Despite dense innervation of the liver parenchyma, little is known about neural regulation of liver function in inflammation. Here, we study vagus nerve control of the liver response to acute inflammation.

**Methods:**

Male C57BL/6 J mice were subjected to either sham surgery, surgical vagotomy, or electrical vagus nerve stimulation followed by intraperitoneal injection of the TLR2 agonist zymosan. Animals were euthanized and tissues collected 12 h after injection. Samples were analyzed by qPCR, RNAseq, flow cytometry, or ELISA.

**Results:**

Hepatic mRNA levels of pro-inflammatory mediators *Ccl2, Il-1β, and Tnf-α* were significantly higher in vagotomized mice compared with mice subjected to sham surgery. Differences in liver *Ccl2* levels between treatment groups were largely reflected in the plasma chemokine (C–C motif) ligand 2 (CCL2) concentration. In line with this, we observed a higher number of macrophages in the livers of vagotomized mice compared with sham as measured by flow cytometry. In mice subjected to electrical vagus nerve stimulation, hepatic mRNA levels of Ccl2, Il1β, and Tnf-α, and plasma CCL2 levels, were significantly lower compared with sham. Interestingly, RNAseq revealed that a key activation marker for hepatic stellate cells (HSC), *Pnpla3*, was the most significantly differentially expressed gene between vagotomized and sham mice. Of note, several HSC-activation associated transcripts were higher in vagotomized mice, suggesting that signals in the vagus nerve contribute to HSC activation. In support of this, we observed significantly higher number of activated HSCs in vagotomized mice as compared with sham as measured by flow cytometry.

**Conclusions:**

Signals in the cervical vagus nerve controlled hepatic inflammation and markers of HSC activation in zymosan-induced peritonitis.

**Supplementary Information:**

The online version contains supplementary material available at 10.1186/s42234-023-00108-3.

## Background

The liver is a key immunological organ (Lindor 2017). The liver immune response is important for physiological homeostasis and excessive inflammation may promote a multitude of liver diseases, including non-alcoholic steatohepatitis (NASH) and liver cancer (Robinson et al. 2016). Accumulating data indicates that neural signals are important regulators of inflammation (Eberhardson et al. 2020). For example, in the cholinergic anti-inflammatory pathway (CAP), efferent signals in the left vagus nerve inhibits release of pro-inflammatory cytokines in the spleen by a mechanism that requires the splenic nerve, acetylcholine-producing choline acetyltransferase (ChAT) + T cells and α7 nicotinic acetylcholine receptor subunit (α7nAChR)-expressing immune cells (Tracey 2002; Olofsson et al. 2012; Meregnani et al. 2011; Rosas-Ballina et al. 2011). In clinical trials, activation of the vagus nerve reduced inflammation in chronic inflammatory diseases such as rheumatoid arthritis (Koopman et al. 2016) and Crohn’s disease (Bonaz et al. 2016).

While the liver parenchyma is extensively innervated, and changes in the innervation have been associated with NASH, the role of neural signals in the liver immune response is not well understood (Adori et al. 2021; Liu et al. 2021). Acute inflammation in the liver triggers the activation of liver resident macrophages, also known as Kupffer cells (KCs). Hepatic stellate cells (HSC) are also activated upon liver damage and are considered important for the progression of liver inflammatory diseases (Stewart et al. 2014; Fujita et al. 2016). Secretion of pro-inflammatory cytokines by KCs and HSCs promotes recruitment of immune cells to the liver and increases liver inflammation (Wen et al. 2021; Baeck et al. 2012). Interestingly, it was reported that activation of afferent fibers of the hepatic branch of the vagus nerve contributes to liver anti-inflammatory responses to hepatitis in rats (Jo et al. 2020) and absence of vagus nerve-derived signals increases pro-inflammatory cytokine levels in the liver during endotoxemia (Borovikova et al. 2000). It has also been reported that cytokine levels in the portal vein are monitored by afferent vagus neurons (Niijima 1996; Wong et al. 2011; Nishio et al. 2017), suggesting the possibility of neural reflex control of liver inflammation. Moreover, there is experimental evidence that loss of vagus nerve signaling or genetic deficiency in the cholinergic α7nAChR promotes activation of KCs in mice fed with a methionine-choline deficient diet (Nishio et al. 2017). However, whether vagus nerve signals modulate also the HSC response during liver inflammation remains poorly explored.

In light of the observations that neural signals regulate liver inflammation (Stewart et al. 2014), we studied vagus nerve control of the liver response in systemic inflammation.

## Methods

### Animals

All procedures with experimental animals were approved by the regional Stockholm Animal Research Ethics Committee (Stockholm, Sweden). Male (age 9–10 wks) C57BL/6 (Charles River Laboratories) and B6.Cg-Tg(RP23-268L19-EGFP)2Mik/J mice were used. Mice were housed under a 12 h light/dark cycle with ad libitum access to food and water.

### Vagotomy and vagus nerve stimulation

The surgery and vagus nerve isolation methods used in this study have been previously described (Caravaca et al. 2019). Briefly, anesthesia was induced using 3% isoflurane with a 1:1 mixture of oxygen and air. Following induction, the isoflurane was lowered and maintained at 1.5%. Mice were then placed on a surgical mat on top of a heating pad and oriented to a supine position. A cervical midline incision was made between the mandible and sternum. Subcutaneous tissues and salivary glands were separated to reveal superficial cervical muscles along the trachea. Further separation of these tissues revealed the carotid artery and the cervical vagus nerve. The left vagus nerve was isolated from the carotid artery and surrounding connective tissues using blunt dissection. For unilateral left cervical vagotomy (VX), the nerve was carefully suspended with forceps and cuts were made above and below the forceps grip to remove a segment of the nerve, as previously described (Caravaca et al. 2019). For vagus nerve stimulation (VNS), the left vagus nerve was suspended on a hook electrode made of 0.25 mm platinum-iridium (Caravaca et al. 2019). Constant current stimulation was applied to the nerve at 1 mA, 250 μs biphasic pulse, 50 μs interphase delay, at 10 Hz for 5 min (Caravaca et al. 2019).

### Inflammatory challenge with zymosan

0.1 mg zymosan (Sigma-Aldrich, #Z4250) was injected intraperitoneally (Underhill et al. 1999; Caravaca et al. 2022). Mice were euthanized by carbon dioxide asphyxiation 12 h after zymosan challenge.

### Liver perfusion, collection, and processing

Livers were perfused through the right chamber of the heart using 10 mL of PBS and the portal vein was cut to limit perfusion circulation to the liver. Briefly, a catheter with a 27G needle attached to a 12 mL syringe containing PBS was inserted in the right heart atrium. For flow cytometry analysis, the left lateral lobe of the liver was collected and weighed. Single-cell suspension of liver tissue were achieved by sharp dissection by scalpel (Swann-Morton, #0501) followed by enzymatic digestion in HBSS (ThermoFisher, #14025050) containing 0.5 mg/mL of collagenase type IV (Sigma-Aldrich, #C4-22-1G) and 0.05 mg/mL of DNAse (Roche, #10104159,001) at 37ºC for 30 min. After digestion, samples were homogenized by passing 5–6 times through an 18G needle attached to 2 mL syringe, and then transferring it to a 70 μm cell strainer placed on a 50 mL Falcon tube. 15 mL of PBS containing 10% FBS and 5 mM EDTA was passed through the cell strainer and samples subsequently centrifuged at 400 G force for 5 min at 4ºC. Then, the pellet was resuspended in red blood lysis buffer (Invitrogen, #00–4333-57) for 3 min at room temperature and 0.6 mL of PBS containing 1% FBS and 5 mM EDTA was added to stop the reaction. Then samples were centrifuged at 400 G force for 5 min at 4ºC to proceed with the staining for flow cytometry.

### Flow cytometry

Liver cells were incubated with anti-mouse CD16/CD32 mouse fragment crystallizable receptor block (Biosciences, #553142) and stained with surface antibodies listed in Supplementary Table 1 and Zombie Aqua™ Fixable Viability Kit (Biolegend, #423101). Samples were then fixed/permeabilized with Foxp3/Transcription Factor Staining Buffer Set (Invitrogen, #00–5523-00) following manufacturer instructions and stained with intracellular antibodies (Supplementary Table 1). Cells were analyzed on Cytek Northern Lights cytometer (Cytek Biosciences, #NL-3000) and with FlowJo software (FlowJo, v10.8.1). Gating strategy is detailed in Figures S1 and S3.

### RNA extraction, cDNA synthesis and real-time RT-PCR

Liver samples were collected and snap frozen in liquid nitrogen and then stored at -80 °C. Snap frozen liver samples were homogenized using tissue lyser in QiaZol. RNA was isolated using RNeasy Mini Kit (Qiagen, #74106) with QiaCube and the RNA concentration was determined using Nanodrop. Reverse transcription was performed using a HighCapacity cDNA Reverse Transcription kit (ThermoFisher, #4368814) using 500 ng of RNA. The mRNA levels were then analyzed using TaqMan Universal PCR Master Mix (ThermoFisher, #4304437) or Power SYBR Green PCR Master Mix (ThermoFisher, #4367659) and normalized to cyclophilin A (Ppia) using specific primers (Supplementary Table 2).

### Cytokine measurement

Blood was collected by cardiac puncture in an EDTA-containing tube and then centrifuged (12 min at 2500 g) to obtain plasma which was subsequently stored at –80 °C. Plasma was thawed and diluted 3 fold in 1% BSA in PBS. CCL2 concentration of diluted plasma was measured by ELISA (R&D, #DY479) according to manufacturer instructions.

### RNA sequencing

Single-read RNA sequencing was performed by Novogene and aligned to the mouse transcriptome (release M31, GRCm39) and quantified using Salmon (Patro et al. 2017). Quantified data was imported into R using tximport (Soneson et al. 2016) and differential expression was assessed using DESeq2 (Love et al. 2014). The code for RNA-seq analysis can be found on https://github.com/ImmunoBioLab/Ahmed2023A.

## Statistics

Results are expressed as mean ± SEM. Differences between groups were analyzed using the Student’s *t* test. *p* < 0.05 was considered significant. Analysis was performed using GraphPad Prism 9.4.1 (GraphPad Software) except for RNA sequencing data which was analyzed as described above.

## Results

### Increased liver inflammation in zymosan-induced peritonitis in vagotomized mice

**Fig. 1:**
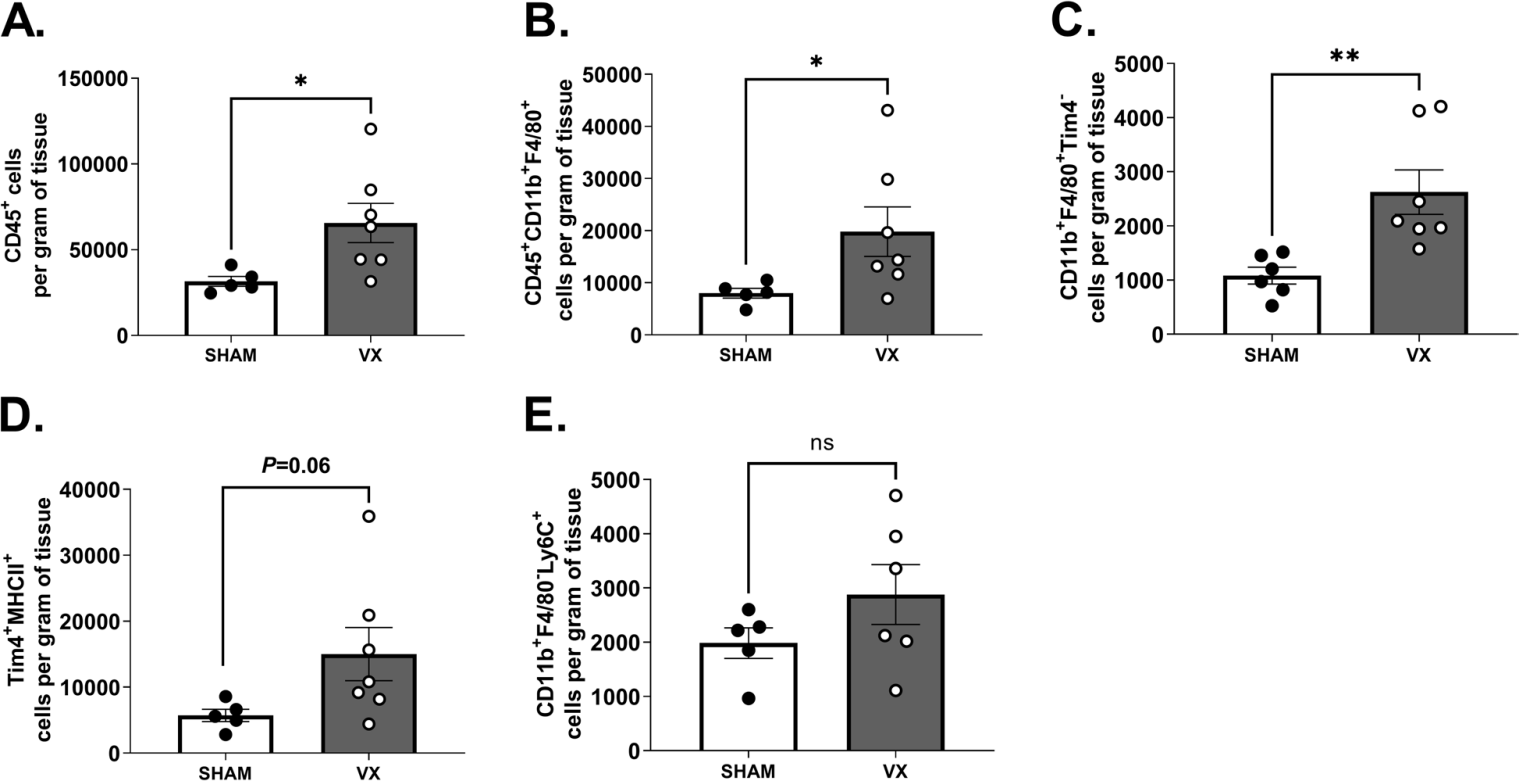
Elevated peritonitis-induced liver leukocyte content in vagotomized mice. Wild-type C57BL/6 J mice were subjected to either left cervical unilateral vagotomy (VX, grey bars) or sham surgery (SHAM, white bars). After 7 days, mice were injected intraperitoneally with zymosan (0.1 mg/mouse) and euthanized 12 h thereafter. Liver homogenates were analyzed by flow cytometry for (**A**) CD45^+^, (**B**) CD45^+^CD11b^+^F4/80^+^, (**C**) CD45^+^CD11b^+^F4/80^+^Tim4^−^, (**D**) CD45^+^CD11b^+^F4/80^+^Tim4^+^MHCII^+^, and (**E**) CD45^+^CD11b^+^F4/80^−^Ly6C^+^ cell subsets. Results were normalized to liver biopsy mass (g). *n* = 6–7 per group. Results are expressed as mean ± SEM. ns = not significant; * *p* < 0.05; ** *p* < 0.01 (two-sided Student´s *t*-test; Welch’s correction was applied as appropriate)

To investigate whether vagus nerve signals regulate liver inflammation in peritonitis, C57BL/6J mice were subjected to left cervical vagotomy (VX) or sham surgery followed by zymosan-induced peritonitis seven days after surgery. Twelve hours after zymosan injection, mice were euthanized and blood and liver samples were collected. Flow cytometry showed that numbers of CD45^+^ leukocytes and CD45^+^CD11b^+^F4/80^+^ macrophages were significantly higher in livers from vagotomized mice compared to sham controls (Fig. 1A-B and Supplementary Figure S1). We also observed that livers from VX mice had more of CD45^+^CD11b^+^F4/80^+^Tim4^−^ macrophages of non-embryonic origin (Fig. 1C) and of CD45^+^CD11b^+^F4/80^+^Tim4^+^MHCII^+^ embryonic resident KCs (Fig. 1D). Moreover, the mean number of CD45^+^CD11b^+^F4/80^−^Ly6C^+^ monocytes in liver homogenates appeared higher in vagotomized than sham mice, but difference was not statistically significant (Fig. 1E).

**Fig. 2:**
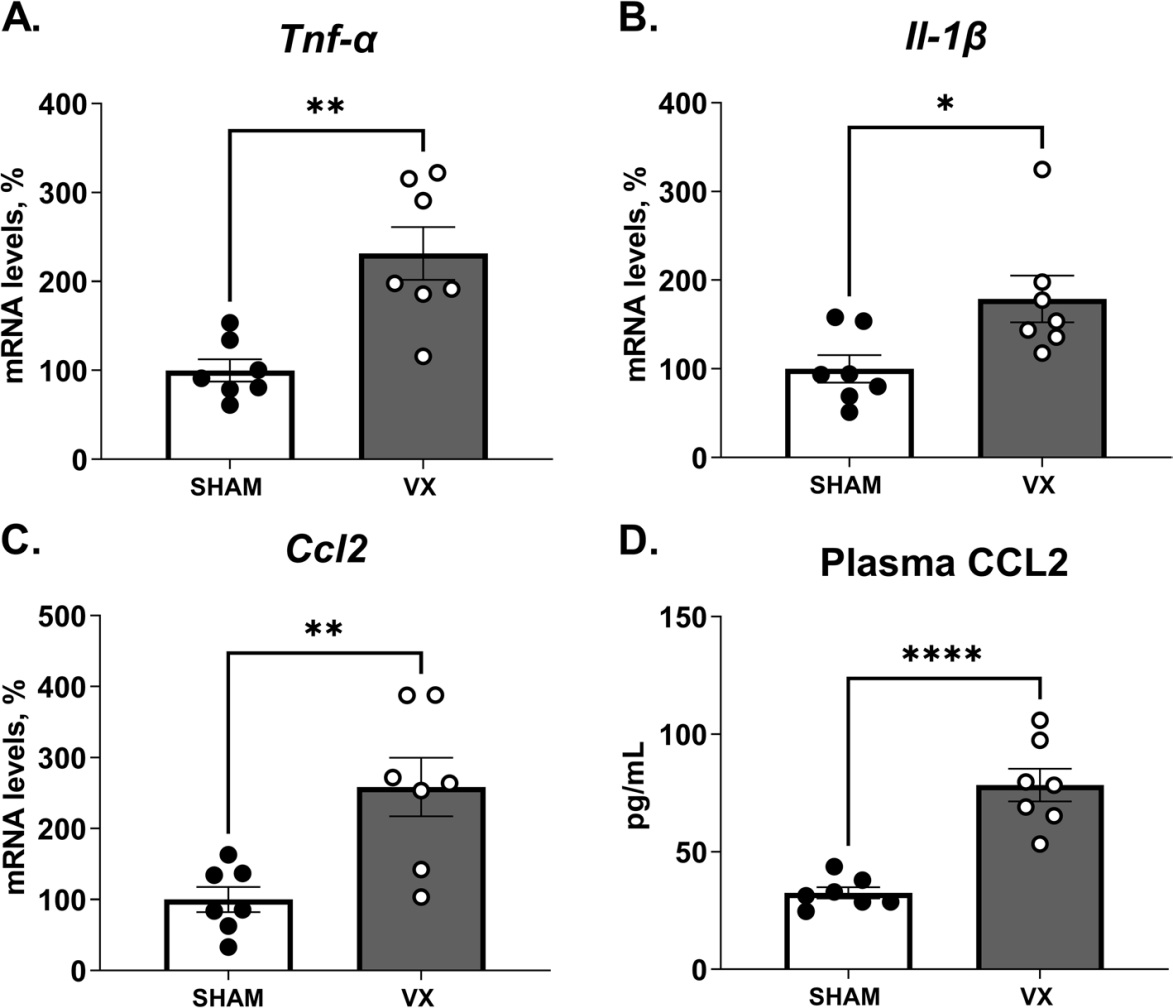
Elevation of peritonitis-induced pro-inflammatory mediators in liver homogenates and blood from vagotomized mice. Wild-type C57BL/6J mice were subjected to either left cervical vagotomy (VX, grey bars) or sham surgery (SHAM, white bars). After 7 days, mice were injected intraperitoneally with zymosan (0.1 mg/mouse) and euthanized after 12 h. Liver and blood samples were collected and analyzed by qPCR and ELISA. (**A**-**C**) Transcripts of pro-inflammatory mediators in liver homogenates were measured by qPCR. Bars show mean ± SEM, dots represent individual mice. Transcript levels were normalized to *Ppia* and expressed relative to SHAM (100%). (**D**) Plasma CCL2 was measured by ELISA. *n* = 7 mice per group. Bars show mean ± SEM, dots represent individual mice. * *p* < 0.05; ** *p* < 0.01; *** *p* < 0.001; **** *p* < 0.0001. (two-sided Student´s *t*-test)

The chemokine CCL2 is an important mediator for immune cell recruitment in hepatic inflammation (Marra and Tacke 2014). TNF-α and IL-1β are the main inflammatory mediators released in liver injury and contribute to hepatocyte cell death, hepatic lipid accumulation, and liver inflammation and hepatotoxicity (Petrasek et al. 2012; Imaeda et al. 2009; Zhao et al. 2020). Levels of inflammation-associated transcripts *Tnf-α*, *Il-1β and Ccl2* were higher in liver homogenates from vagotomized mice as compared with sham (Fig. 2A-C), as was plasma CCL2 as determined by ELISA (Fig. 2D). *Itgam* (CD11b) transcripts in liver homogenates were elevated in vagotomized as compared with sham treated mice (Supplementary Figure S2 A). These observations indicate that signals in the vagus nerve control aspects of the liver inflammatory response in peritonitis.

### Electrical vagus nerve stimulation reduced liver inflammation in zymosan-induced peritonitis

**Fig. 3:**
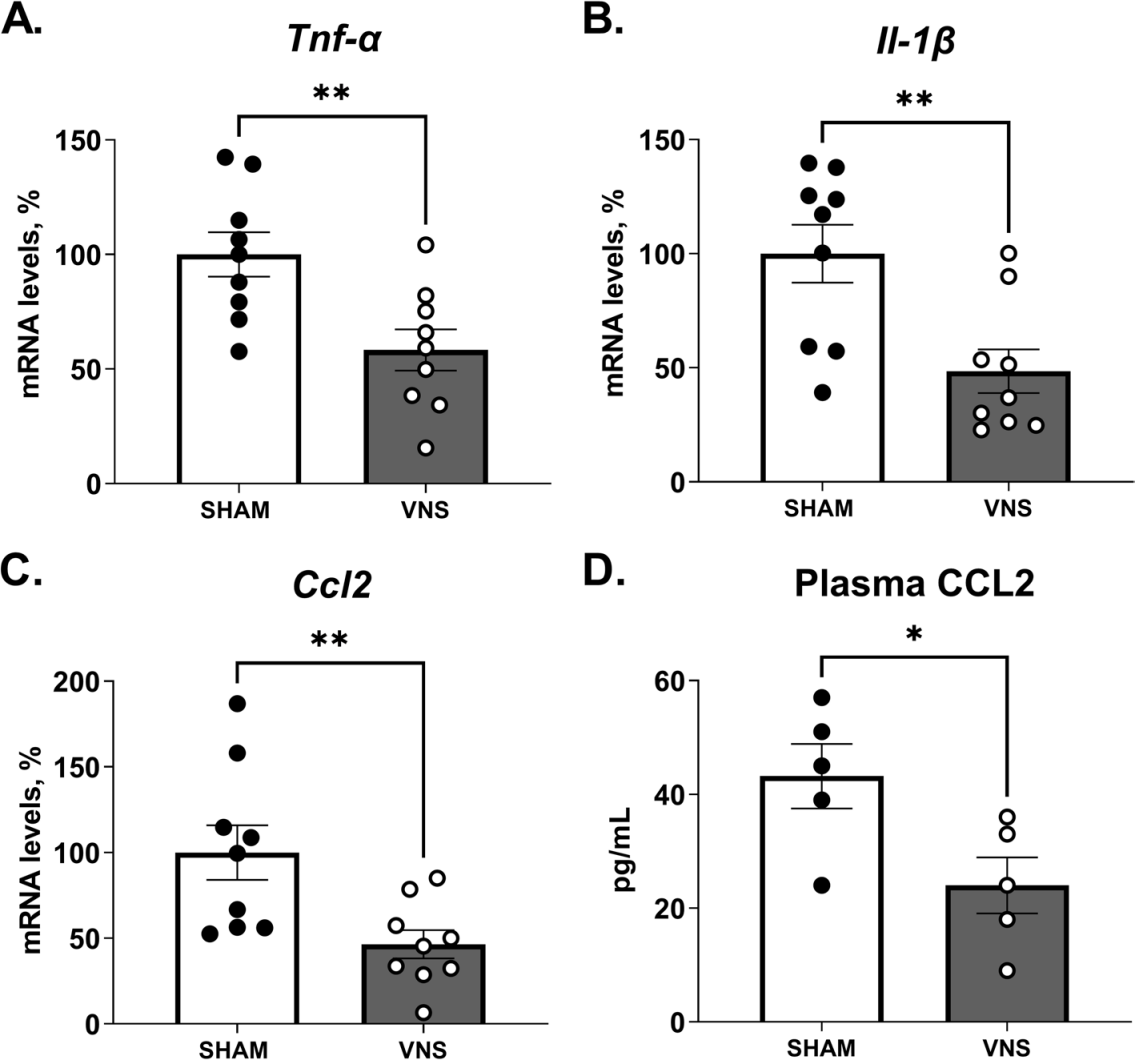
Reduced levels of peritonitis-induced pro-inflammatory mediators in livers from mice subjected to electrical vagus nerve stimulation. Wild-type C57BL/6J mice were subjected to either left cervical unilateral vagus nerve stimulation (VNS, grey bars) or sham surgery (SHAM, white bars) followed by intraperitoneal injection of zymosan (0.1 mg/mouse) 1 h thereafter. Liver and blood samples were collected 12 h after zymosan injection and analyzed by qPCR and ELISA. (**A**-**C**) Transcripts of pro-inflammatory mediators were measured in liver homogenates by qPCR (two independent experiments, *n* = 5 or 4 per group, respectively). Bars show mean ± SEM, dots represent individual mice. Transcript levels were normalized to *Ppia* and expressed relative to SHAM (100%). (**D**) Plasma CCL2 was measured by ELISA (*n* = 5 mice per group). Bars show mean ± SEM, dots represent individual mice. * *p* < 0.05; ** *p* < 0.01 (two-sided Student´s *t*-test)

Next, mice were subjected to VNS or sham surgery followed by zymosan-induced peritonitis one hour thereafter. Twelve hours after zymosan injection, mice were euthanized, and blood and liver samples were collected. Levels of inflammation-associated transcripts *Tnf-α*, *Il-1β and Ccl2* were lower in liver homogenates from mice subjected to VNS as compared with sham (Fig. 3A-C), as was plasma CCL2 (Fig. 3D). While *Itgam* was not significantly regulated by vagus nerve activation in this setting, transcript levels of *Itgax* (Cd11c) and *Adgre1* (F4/80) were significantly reduced in the VNS-treated group as compared with sham (Supplementary Fig. 2 E–F). These results further support the hypothesis that signals in the vagus nerve are involved in aspects of the liver response to acute inflammation.

### Activation of hepatic stellate cells in peritoneal inflammation controlled by the vagus nerve

**Fig. 4:**
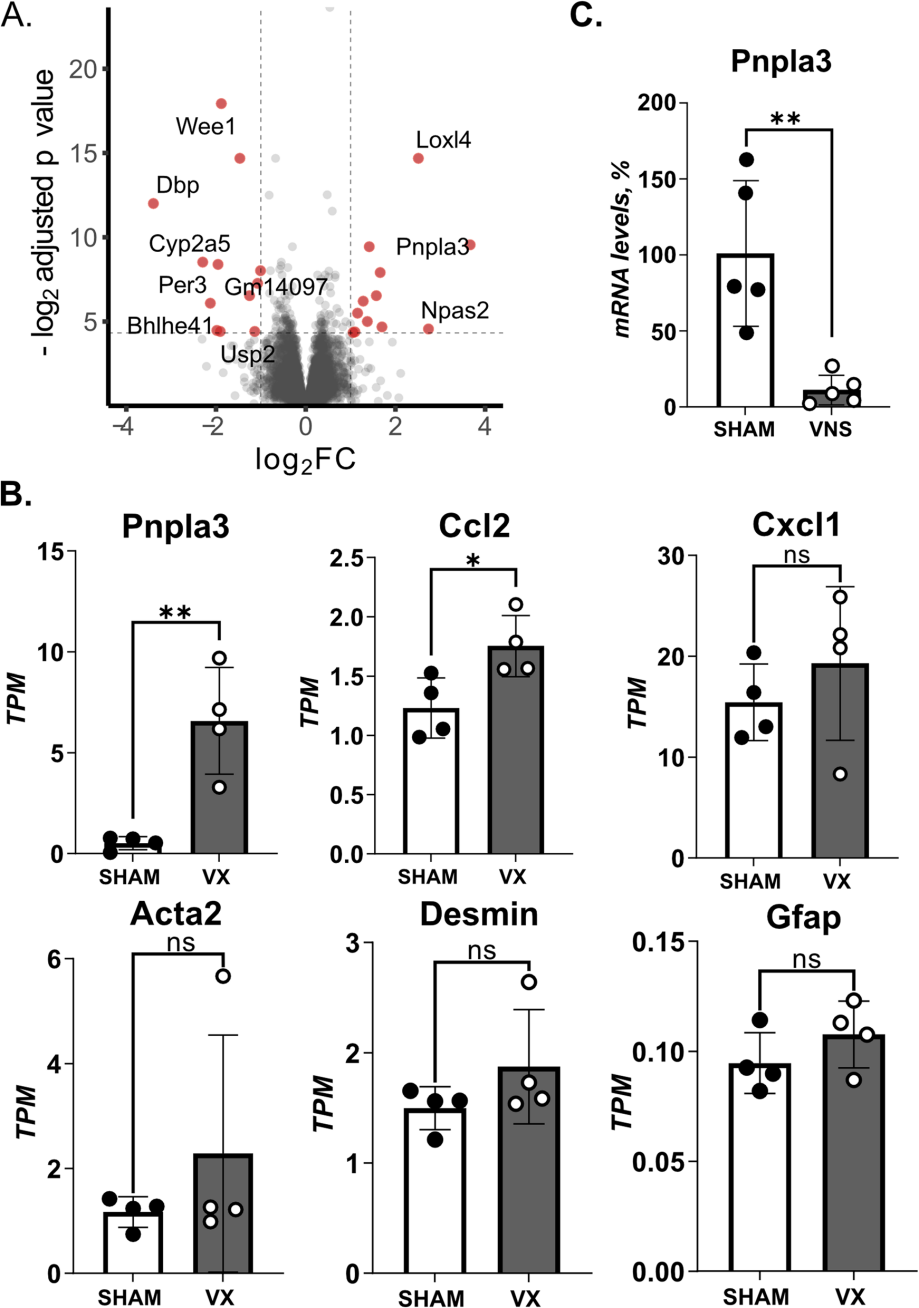
HSC activation-markers in inflammation controlled by the vagus nerve. (**A**-**B**) Wild-type C57BL/6J mice were subjected to either left cervical unilateral vagotomy (VX) (*n* = 4) or sham surgery (*n* = 4). After 7 days, mice were injected with zymosan (0.1 mg/mouse) and euthanized after 12 h. Liver biopsies were collected and analyzed by RNA sequencing. (**A**) Volcano plot of differentially regulated genes between VX and sham mice. Red color indicates significant fold change (FC) outside limits shown by dashed lines (FC ≥ 1, FDR-adjusted *p* < 0.05) (**B**) Levels of select inflammation- and hepatic stellate cell (HSC)-associated transcripts were plotted. (**C**) Wild-type C57BL/6J mice were subjected to either left cervical unilateral vagus nerve stimulation (VNS, grey bars) or sham surgery (SHAM, white bars) followed by intraperitoneal injection of zymosan (0.1 mg/mouse) 1 h thereafter. Liver and blood samples were collected 12 h after zymosan injection and analyzed by qPCR (*n* = 5 per group). Bars show mean ± SEM, dots represent individual mice. PNPLA3 mRNA level were normalized to *Ppia* and expressed relative to SHAM (100%). * *p* < 0.05; ** *p* < 0.01 (two-sided Student´s *t*-test)

We proceeded to analyze the effect of vagus nerve signals on the whole liver transcriptome in acute inflammation. As in our previous experiments reported here, mice were subjected to left cervical unilateral VX followed by seven days rest with subsequent zymosan-induced peritonitis. Livers were collected 12 h after zymosan-injection and homogenates analyzed by RNA sequencing. In agreement with the observations in Figs. 1 and 2, levels of several inflammation-associated transcripts were controlled by signals in the vagus nerve (Fig. 4). These observations further support that the liver response to zymosan-induced acute inflammation is controlled by signals in the vagus nerve.

Of note, liver patatin-like phospholipase domain–containing 3 (*Pnpla3)* showed the largest effect size among the detected transcripts in VX as compared with sham-treated mice with zymosan-induced peritonitis (Fig. 4A-B). This is interesting, because *Pnpla3* is required for HSC activation (Bruschi et al. 2017) and a genetic variant of *PNPLA3* predisposes for development of non-alcoholic fatty liver disease (NAFLD) (Romeo et al. 2008), the pathogenesis of which includes HSC activation (Loomba et al. 2021). Accordingly, we proceeded to analyze the effect by VX on transcripts associated with HSC activation in the context of zymosan-induced peritonitis. We observed that the mean levels of HSC-activation-associated transcripts *Acta2*, *Desmin*, and *Gfap* appeared higher in liver homogenates from vagotomized mice as compared to sham, but the differences were not statistically significant (Fig. 4B). Several transcripts with connection to regulation of liver pathophysiology in inflammation and fibrosis were significantly different between groups (Supplementary Fig. 4). mRNA levels of *Pnpla3* were lower in VNS-treated as compared with sham-treated mouse liver homogenates (Fig. 4C).

**Fig. 5:**
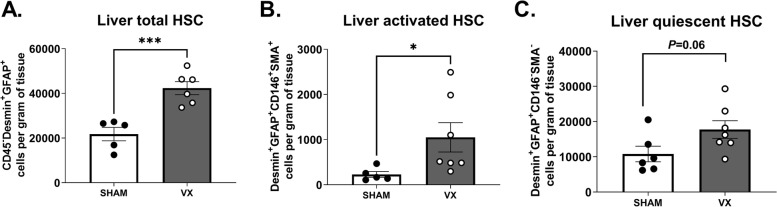
Higher activation of hepatic stellate cells in acute inflammation in vagotomized mice. Wild-type C57BL/6J mice were subjected to either left cervical unilateral vagotomy (VX, grey bars) or sham surgery (SHAM, white bars). After 7 days, mice were injected intraperitoneally with zymosan (0.1 mg/mouse) and euthanized 12 h thereafter. Liver homogenates were analyzed for (**A**) CD45^−^Desmin^+^GFAP^+^ HSCs, (**B**) CD45^−^Desmin^+^GFAP^+^CD146^+^SMA^+^ activated HSCs, and (**C**) CD45^−^Desmin^+^GFAP^+^CD146^−^SMA.^−^ quiescent HSCs, and normalized by liver biopsy mass (g). *n* = 6–7 per group. Results are shown as mean ± SEM. ns = not significant; * *p* < 0.05, *** *p* < 0.001 (two-sided Student *t*-test; Welch’s test correction was applied when variances were significantly different)

To investigate the observations on HSC-activation-associated transcripts further, we subjected another set of mice to left cervical unilateral VX or sham surgery followed by zymosan-induced peritonitis 7 days thereafter. HSC activation was analyzed in liver homogenates using flow cytometry (Supplementary Fig. 3). We observed significantly higher numbers of CD45^−^Desmin^+^GFAP^+^ HSCs and CD45^−^ CD146^+^ Desmin^+^GFAP^+^SMA^+^ activated HSCs per gram of tissue in VX mice as compared with sham, but no significant difference between groups in the number of CD45^−^ CD146^−^Desmin^+^GFAP^+^SMA^−^ quiescent HSCs per gram tissue (Fig. 5), corroborating that vagus nerve signals regulated HSC activation and number in acute inflammation.

## Discussion

Here, we found that signals in the cervical vagus nerve controlled hepatic inflammation in zymosan-induced peritonitis. The loss of vagus nerve signals exacerbated the expression of hepatic pro-inflammatory cytokines, while electrical activation of the vagus nerve reversed this effect—consistent with the established role of cholinergic anti-inflammatory pathway in other tissues like the spleen. Furthermore, interruption of vagus nerve signals increased the number of macrophages and activated HSC in the liver.

The observation here that loss of vagus nerve signals promoted an increase in the number of liver macrophages during acute inflammation indicates that the vagus nerve is involved in the control of liver macrophages in acute inflammation. In line with this, the loss of vagus nerve signals also induced hepatic mRNA expression of pro-inflammatory cytokines. The increased expression of *Ccl2* in vagotomized mice may contribute to the higher number of liver macrophages by increasing macrophage recruitment. In support of our data, Nishio and colleagues (Nishio et al. 2017) found an increase in CCL2 in vagotomized mice in diet-induced NASH, a chronic inflammatory condition. Together, these findings support that signals in the vagus nerve may regulate both acute and diet-induced chronic inflammation in the liver.

Interestingly, the most differentially expressed gene in vagotomized mice was *Pnpla3*. It was previously shown that expression of *PNPLA3* increases during the early phases of human HSC activation and remains elevated in activated HSCs (Bruschi et al. 2017). However, the role of PNPLA3 in transforming growth factor (TGF)-mediated activation of human HSCs is not fully understood (Rady et al. 2021). Moreover, activated HSCs contribute to liver injury during acute inflammation (Stewart et al. 2014; Fujita et al. 2016) and deletion of HSCs protects the liver from damage progression (Stewart et al. 2014), indicating an important role of these cells in liver inflammation. Our RNAseq data showed increased expression of other activated HSC associated transcripts in vagotomized mice, and qPCR showed that VNS reduced *Pnpla3*. Accordingly, it is reasonable to hypothesize that signals in the vagus nerve may be required to maintain hepatic stellate cells in the quiescent state. In support of this notion, we observed an increase in numbers and activation of HSCs in vagotomized mice. We speculate that the signs of increased HSC activation associated with the loss of vagus nerve signal may contribute to the higher intensity of inflammation observed in these mice. As CCL2 was reported to be secreted by HSC upon acute hepatic damage (Seki et al. 2007), one possible mechanism for increased liver inflammation is that increased CCL2 release by activated HSC in the absence of vagus nerve derived signals may promote immune cell recruitment. Alternatively, since we observed increased number of macrophages after vagotomy, these cells could be the source of elevated CCL2, as they also contribute to CCL2 production during hepatic injury (Mandrekar et al. 2011).

Activation of HSCs is important in the pathogenesis of NASH. Intriguingly, nerve endings are found in the perisinusoidal space in the livers of humans, cats, and guinea pigs (Ueno et al. 2004) where KC and HSC are also found, but whether neural signals regulate inflammation in the KC niche is unknown. Here, we observed a higher number of both HSCs and embryonic KCs in vagotomized mice. KCs interact closely with hepatic stellate cells (HSCs), which are essential for maintaining the KC niche (Bonnardel et al. 2019). It is tempting to speculate that neural signals contribute to maintaining the KC niche and regulate both HSCs and KCs under inflammatory conditions. It will be exciting to explore these hypotheses further in relevant experimental models.

A consideration for the interpretation of the RNAseq data and qPCR is that many genes are expressed by a multitude of cells in the liver, and it will be important to further investigate the cell-specific expression of the involved mediators, including PNPLA3.

Vagus nerve activation using implanted devices is already in clinical use, and recent clinical trials of VNS for treatment of excessive inflammation show promising results in rheumatoid arthritis and Crohn’s disease (Koopman et al. 2016; Bonaz et al. 2016). As our mechanistic understanding of the neural regulation of liver inflammation develops further, it is conceivable that targeting relevant neural circuits in the liver may be a strategy for controlling excessive inflammation in select liver diseases.

## Conclusion

The observations here indicate that signals in the vagus nerve regulate liver inflammation, including the hepatic content of macrophages and HSC activity in zymosan-induced peritonitis. It will now be important to explore the contribution of neural regulation of inflammation in the pathophysiology of liver inflammation in NAFLD and NASH.

## Supplementary Information

**Additional file 1: Figures S1**. Representative plots of flow cytometry gating for monocytes and macrophages in liver homogenates. (A) From left to right: cells were selected based on size and granularity (FSC, SSC), identification of singlets, viability by Zombie Aqua staining, and CD45 immunostaining. Subsequently, (B) CD45 + F4/80 + CD11b + liver macrophages, (C) CD45 + F4/80 + CD11b + Tim4 + MHCII + embryonic KCs and CD45 + F4/80 + CD11b + Tim4- non-embryonic macrophages, and (D) CD45 + F4/80-CD11b + Ly6C + monocytes were identified. Fluorescence minus one (FMO) controls were used as reference for the positive and negative gates. **Figure S2.** Vagus nerve signals regulated the mRNA levels of immune cell markers in the liver during zymosan-induced peritonitis. (A-C) Hepatic mRNA levels of immune cell markers in wild-type C57BL/6J mice subjected to left cervical unilateral vagotomy (VX, grey bars) or sham surgery (SHAM, white bars) followed by intraperitoneal injection of zymosan (0.1 mg/mouse) 7 days thereafter. (D-F) Hepatic mRNA levels of immune cell markers from wild-type mice subjected to left cervical unilateral vagus nerve stimulation (VNS, grey bars) or sham surgery (SHAM, white bars) followed by intraperitoneal injection of zymosan (0.1 mg/mouse) 1 h thereafter. Liver samples were collected 12 h after zymosan injection and homogenates analyzed by qPCR. *n* = 7–9 mice per group from two independent experiments. Mean ± SEM was plotted. ns = not significant; * *p* < 0.05, ** *p* < 0.01 (two-sided Student´s *t*-test). **Figure S3.** Representative plots of flow cytometry gating for hepatic stellate cells in liver homogenates. Cells were gated from the CD45^−^ population described in Figure S1A. (A) Cells were selected based on the expression of CD146, Desmin, GFAP and SMA for the detection of CD45^−^CD146^+^Desmin^+^GFAP^+^SMA^+^ activated HSC (aHSC) and CD45^−^CD146^+^Desmin^+^GFAP^+^SMA^+^ quiescent HSC (qHSC). (B) CD45^−^ Desmin^+^GFAP^+^ HSC were identified. Fluorescence minus one (FMO) controls were used as reference for the positive and negative gates. **Figure S4.** The top 10 most significantly differentially expressed genes. Wild-type C57BL/6 J mice subjected to left cervical unilateral vagotomy (VX, grey bars) or sham surgery (SHAM, white bars). After 7 days, mice were injected with zymosan (0.1 mg/mouse) and euthanized after 12 h. Liver biopsies were collected and analyzed by RNA sequencing. Levels of the ten most differentially expressed transcripts were plotted. *n* = 4 per group. Bars show mean ± SEM, dots represent individual mice. * *p* < 0.05; ** *p* < 0.01 (two-sided Student´s *t*-test). **Supplementary Table 1.** Antibodies for surface and intracellular staining of liver cells for flow cytometry. **Supplementary Table 2.** Primer Sequences for Real-Time PCR.

## Data Availability

R scripts are found at https://github.com/ImmunoBioLab/Ahmed2023A. Data is available from the corresponding author.

## Abbreviations

CAP: Cholinergic anti-inflammatory pathway
CCL2: Chemokine (C–C motif) ligand 2
GO: Gene Onthology
GSEA: Gene Set Enrichment Analysis
HSC: Hepatic stellate cell
KC: Kupffer cell
NAFLD: Non-alcoholic fatty liver disease
NASH: Non-alcoholic steatohepatitis
TGF: Transforming growth factor
VNS: Vagus nerve stimulation
VX: Vagotomy
α7nAChR: α7 Nicotinic acetylcholine receptor subunit
