# Supplementary material for: Hepatic stellate cell activation markers are regulated by the vagus nerve in systemic inflammation
Source: Bioelectron Med. 2023 Mar 31;9:6. doi: 10.1186/s42234-023-00108-3 (PMC10064698; doi:10.1186/s42234-023-00108-3)
Supplement: Supplementary file 1 — Additional file 1: Figures S1. Representative plots of flow cytometry gating for monocytes and macrophages in liver homogenates. (A) From left to right: cells were selected based on size and granularity (FSC, SSC), identification of singlets, viability by Zombie Aqua staining, and CD45 immunostaining. Subsequently, (B) CD45 + F4/80 + CD11b + liver macrophages, (C) CD45 + F4/80 + CD11b + Tim4 + MHCII + embryonic KCs and CD45 + F4/80 + CD11b + Tim4- non-embryonic macrophages, and (D) CD45 + F4/80-CD11b + Ly6C + monocytes were identified. Fluorescence minus one (FMO) controls were used as reference for the positive and negative gates. Figure S2. Vagus nerve signals regulated the mRNA levels of immune cell markers in the liver during zymosan-induced peritonitis. (A-C) Hepatic mRNA levels of immune cell markers in wild-type C57BL/6J mice subjected to left cervical unilateral vagotomy (VX, grey bars) or sham surgery (SHAM, white bars) followed by intraperitoneal injection of zymosan (0.1 mg/mouse) 7 days thereafter. (D-F) Hepatic mRNA levels of immune cell markers from wild-type mice subjected to left cervical unilateral vagus nerve stimulation (VNS, grey bars) or sham surgery (SHAM, white bars) followed by intraperitoneal injection of zymosan (0.1 mg/mouse) 1 h thereafter. Liver samples were collected 12 h after zymosan injection and homogenates analyzed by qPCR. n = 7–9 mice per group from two independent experiments. Mean ± SEM was plotted. ns = not significant; * p < 0.05, ** p < 0.01 (two-sided Student´s t-test). Figure S3. Representative plots of flow cytometry gating for hepatic stellate cells in liver homogenates. Cells were gated from the CD45− population described in Figure S1A. (A) Cells were selected based on the expression of CD146, Desmin, GFAP and SMA for the detection of CD45−CD146+Desmin+GFAP+SMA+ activated HSC (aHSC) and CD45−CD146+Desmin+GFAP+SMA+ quiescent HSC (qHSC). (B) CD45− Desmin+GFAP+ HSC were identified. Fluorescence minus one (FMO) controls wer [file 42234_2023_108_MOESM1_ESM.docx]

**Additional file 1**


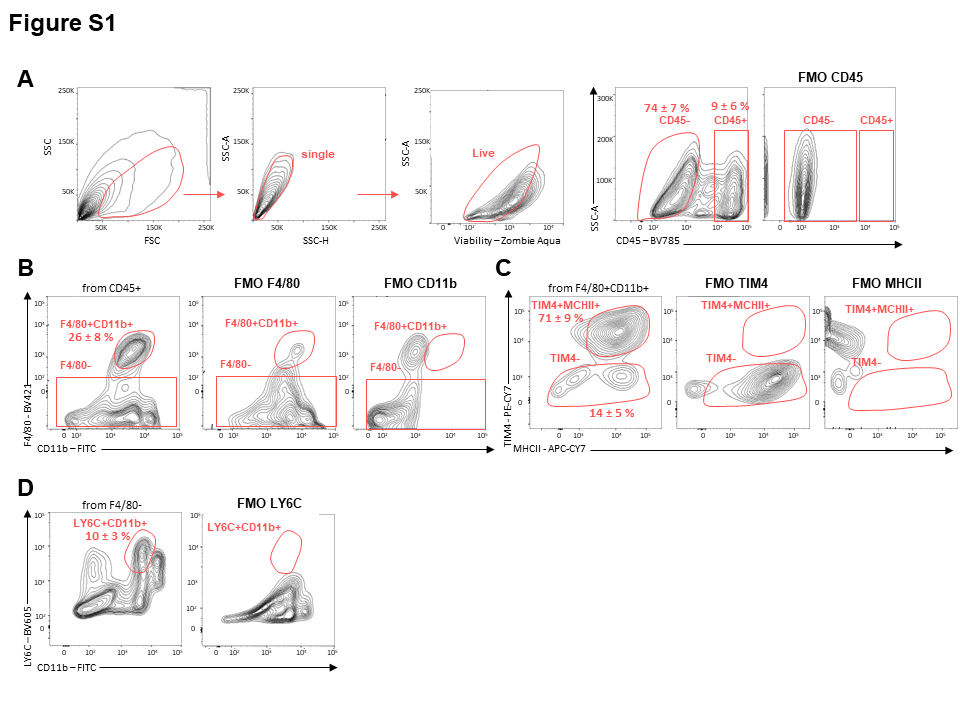


**Figures S1: Representative plots of flow cytometry gating for monocytes and macrophages in liver homogenates. (A)** From left to right: cells were selected based on size and granularity (FSC, SSC), identification of singlets, viability by Zombie Aqua staining, and CD45 immunostaining. Subsequently, **(B)** CD45+F4/80+CD11b+ liver macrophages, **(C)** CD45+F4/80+CD11b+Tim4+MHCII+ embryonic KCs and CD45+F4/80+CD11b+Tim4- non-embryonic macrophages, and **(D)** CD45+F4/80-CD11b+Ly6C+ monocytes were identified. Fluorescence minus one (FMO) controls were used as reference for the positive and negative gates.


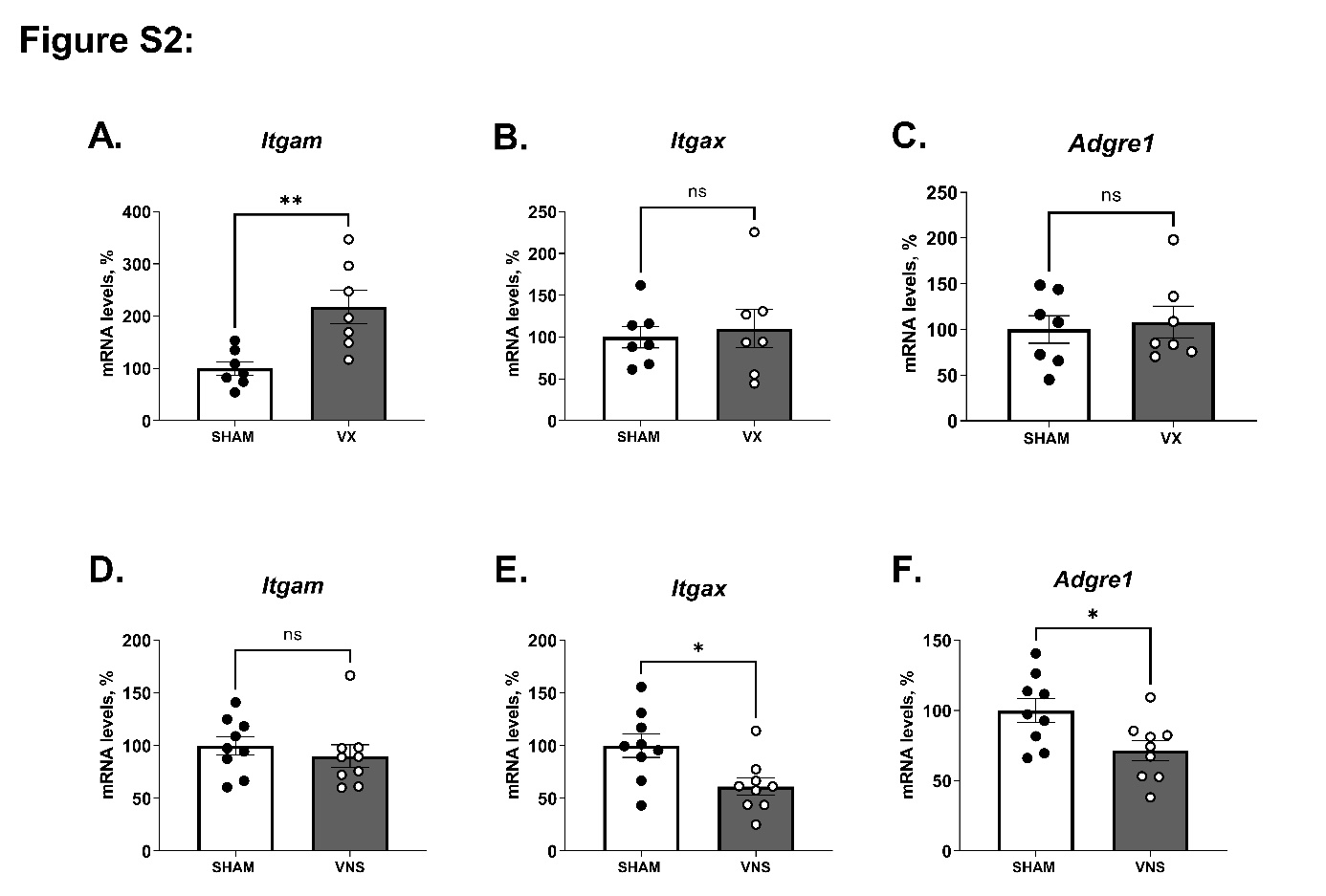


**Figure S2: Vagus nerve signals regulated the mRNA levels of immune cell markers in the liver during zymosan-induced peritonitis. (A-C)** Hepatic mRNA levels of immune cell markers in wild-type C57BL/6J mice subjected to left cervical unilateral vagotomy (VX, grey bars) or sham surgery (SHAM, white bars) followed by intraperitoneal injection of zymosan (0.1 mg/mouse) 7 days thereafter. **(D-F)** Hepatic mRNA levels of immune cell markers from wild-type mice subjected to left cervical unilateral vagus nerve stimulation (VNS, grey bars) or sham surgery (SHAM, white bars) followed by intraperitoneal injection of zymosan (0.1 mg/mouse) 1 h thereafter. Liver samples were collected 12 h after zymosan injection and homogenates analyzed by qPCR. n = 7-9 mice per group from two independent experiments. Mean ± SEM was plotted. ns = not significant; * p < 0.05, ** p < 0.01 (two-sided Student´s *t*-test).


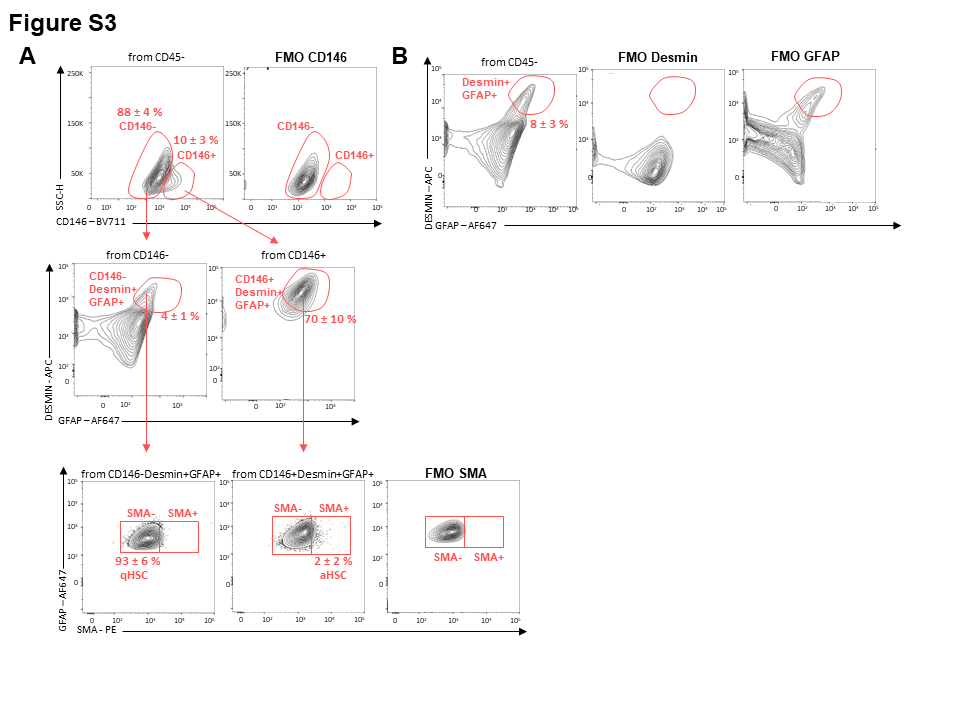


**Figure S3. Representative plots of flow cytometry gating for hepatic stellate cells in liver homogenates.** Cells were gated from the CD45^-^ population described in Figure S1A. **(A)** Cells were selected based on the expression of CD146, Desmin, GFAP and SMA for the detection of CD45^-^CD146^+^Desmin^+^GFAP^+^SMA^+^ activated HSC (aHSC) and CD45^-^CD146^+^Desmin^+^GFAP^+^SMA^+^ quiescent HSC (qHSC). **(B)** CD45^-^ Desmin^+^GFAP^+^ HSC were identified. Fluorescence minus one (FMO) controls were used as reference for the positive and negative gates.

**
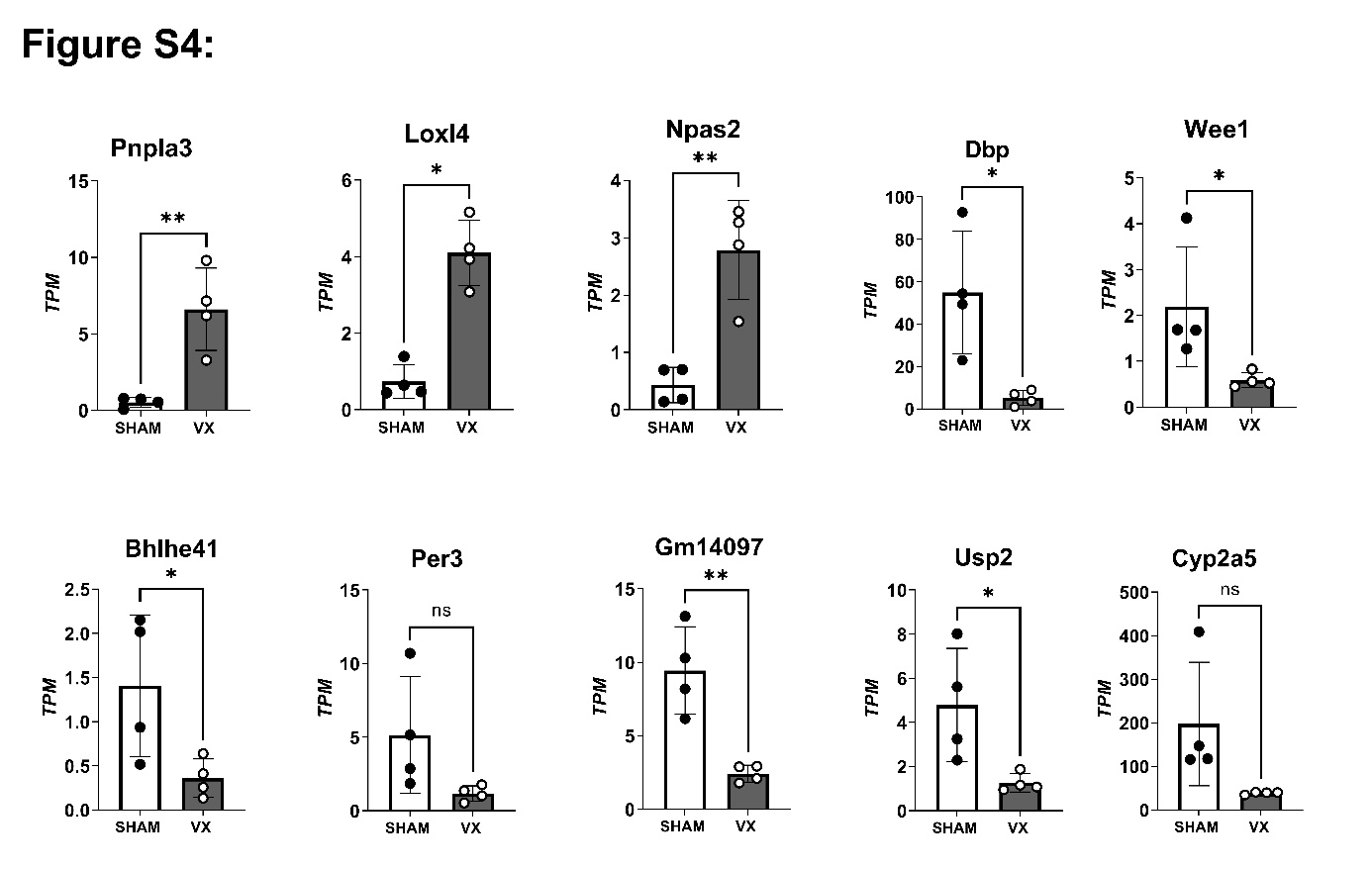
Figure S4: The top 10 most significantly differentially expressed genes.** Wild-type C57BL/6J mice subjected to left cervical unilateral vagotomy (VX, grey bars) or sham surgery (SHAM, white bars). After 7 days, mice were injected with zymosan (0.1 mg/mouse) and euthanized after 12 h. Liver biopsies were collected and analyzed by RNA sequencing. Levels of the ten most differentially expressed transcripts were plotted. n = 4 per group. Bars show mean±SEM, dots represent individual mice. * p < 0.05; ** p < 0.01 (two-sided Student´s *t*-test).

**Supplementary Table 1. Antibodies for surface and intracellular staining of liver cells for flow cytometry.**

| **Antibody** | **Clone** | **Manufacturer** | **Reference** | **Dilution (in 1x PBS)** |
| --- | --- | --- | --- | --- |
| **Surface antibodies** | | | | |
| Anti-mouse CD45 | 30-F11 | Biolegend | 103149 | 1/200 |
| Anti-mouse CD11b | M1/70 | BD Biosciences | 557396 | 1/200 |
| anti-mouse F4/80 | BM8 | Biolegend | 123132 | 1/200 |
| anti-mouse Tim4 | RMT4-54 | Biolegend | 130010 | 1/200 |
| anti-mouse MHCII | M5/114.15.2 | Biolegend | 107628 | 1/200 |
| anti-mouse Ly6C | AL-21 | BD Bioscience | 563011 | 1/200 |
| anti-mouse CD146 | ME-9F1 | BD Bioscience | 740827 | 1/200 |
| **Intracellular antibodies** | | | | |
| anti-mouse/human SMA | 1A4 | LS Bio | LS‑C124868 | 1/200 |
| anti-mouse/human Desmin | OTI4G1 | Novus Biologicals | NBP2-70565APC | 1/200 |
| anti-mouse/human GFAP | 2.2B10 | Invitrogen | 51979282 | 1/200 |

**Supplementary Table 2. Primer Sequences for Real-Time PCR**

| **Gene** | **Forward primers (5ʹ - 3ʹ)** | **Reverse primer (5ʹ - 3ʹ)** |
| --- | --- | --- |
| ***Ccl2*** | **CAGATGCAGTTAACGCCCCA** | **TGAGCTTGGTGACAAAAACTACAG** |
| ***Tnf-α*** | **CCCTCACACTCAGATCATCTTCT** | **GCTACGACGTGGGCTACAG** |
| ***Il1b*** | **GCAACTGTTCCTGAACTCAACT** | **ATCTTTTGGGGTCCGTCAACT** |
| ***Adgre1* (F4/80)** | **CTTTGGCTATGGGCTTCCAGTC** | **GCAAGGAGGACAGAGTTTATCGTG** |
| ***Itgam*** | **CAGACAGGAAGTAGCAGCTCCT** | **CTGGTCATGTTGATGAAGGTGCT** |
| ***Itgax*** | **GGCTATCAAGCATGTCATAACAGAAC** | **CCCCTTGTTTTCTCCCATCAG** |
| ***Ppia* (Cyclophilin A)** | **GAT GAG AAC TTC ATC CTA AAG CAT ACA** | **TCA GTC TTG GCA GTG CAG ATA AA** |

All primer design was based on mouse sequences. Ccl2 - Chemokine (C-C motif) ligand 2; Tnf-α - tumor necrosis factor alpha; Il1β - Interleukin 1 Beta; Adgre1(F4/80) - Adhesion G Protein-Coupled Receptor E1; Itgam (Cd11b) - Integrin Subunit Alpha M; Itgax (Cd11c) - Integrin Subunit Alpha X and Ppia (Cyclophilin A) - peptidylprolyl isomerase A.
